# The Design of 3D-Printed Lattice-Reinforced Thickness-Varying Shell Molds for Castings

**DOI:** 10.3390/ma11040535

**Published:** 2018-03-30

**Authors:** Haolong Shangguan, Jinwu Kang, Jihao Yi, Xiaochuan Zhang, Xiang Wang, Haibin Wang, Tao Huang

**Affiliations:** 1 Key Laboratory for Advanced Materials Processing Technology, School of Materials Science and Engineering, Tsinghua University, Beijing 100084, China; shangghl@163.com (H.S.); 13881717307@163.com (J.Y.); zhang-xc16@mails.tsinghua.edu.cn (X.Z.); wangxian17@mails.tsinghua.edu.cn (X.W.); 2 School of Computer Science, Peking University, Beijing 100871, China; beach@pku.edu.cn; 3 Beijing DRUCK Technology Development Co. Ltd., Beijing 100029, China; taokonstanz@126.com

**Keywords:** 3D printing, computer-aided design, casting, heat transfer, solidification

## Abstract

3D printing technologies have been used gradually for the fabrication of sand molds and cores for castings, even though these molds and cores are dense structures. In this paper, a generation method for lattice-reinforced thickness-varying shell molds is proposed and presented. The first step is the discretization of the STL (Stereo Lithography) model of a casting into finite difference meshes. After this, a shell is formed by surrounding the casting with varying thickness, which is roughly proportional to the surface temperature distribution of the casting that is acquired by virtually cooling it in the environment. A regular lattice is subsequently constructed to support the shell. The outside surface of the shell and lattice in the cubic mesh format is then converted to STL format to serve as the external surface of the new shell mold. The internal surface of the new mold is the casting’s surface with the normals of all of the triangles in STL format reversed. Experimental verification was performed on an Al alloy wheel hub casting. Its lattice-reinforced thickness-varying shell mold was generated by the proposed method and fabricated by the binder jetting 3D printing. The poured wheel hub casting was sound and of good surface smoothness. The cooling rate of the wheel hub casting was greatly increased due to the shell mold structure. This lattice-reinforced thickness-varying shell mold generation method is of great significance for mold design for castings to achieve cooling control.

## 1. Introduction

The 3D printing technology, which can create complicated structures and improve product quality, has greatly promoted the manufacturing industry [1,2,3,4,5]. Fenton et al. proposed a topology-based grammatical evolution (GE) algorithm to create practical 3D structures [6]. Dutta and Froes investigated a light structure with a considerable number of holes for a seat buckle and compared it to the traditional solid wall structure [7]. Wood and Ravi integrated many parts into a single part through 3D printing technology, which can improve dimensional accuracy [8].

3D printing technology has already been applied in the foundry industry [9,10,11,12,13]. Kang et al. presented new mold structures, which contain a mold shell, with functional structures and an enforced skeleton [14,15,16]. Due to its unique structural features, the model construction of this type of molds is hard and time-consuming. Alok et al. [17] presented a design method for multi-piece molds, although the molds were still composed of dense shapes. Chen et al. [18,19] proposed a design method, which creates hollowed-out model structures with a truss structure; however, no shell was involved, and it could not generate the cavity for castings. There is no design software for this type of lattice-reinforced shell mold. Therefore, it is necessary to develop a generation method and tool for the lattice-reinforced thickness-varying shell mold model for 3D printing.

In the present research, a generation method for the lattice-reinforced thickness-varying shell mold is presented. Subsequent experimental verification was performed on an Al alloy wheel hub casting.

## 2. Generation Method

**Figure 1:**
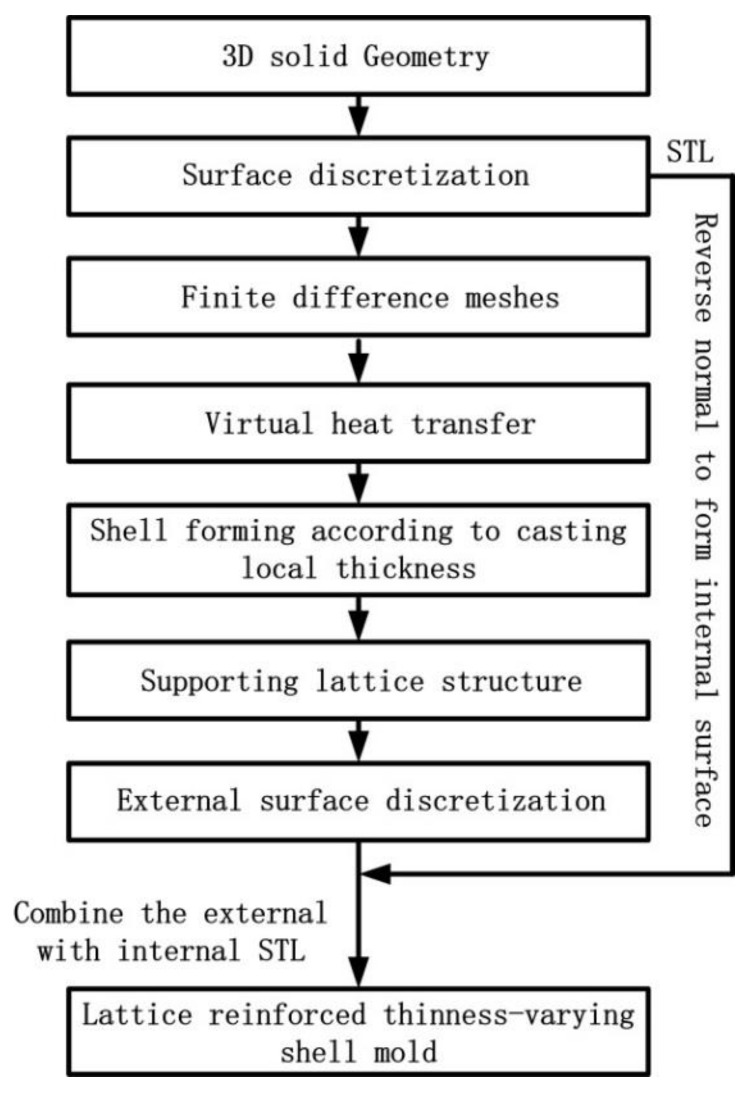
The process for generating the lattice-reinforced thickness-varying shell mold.

**Figure 2:**
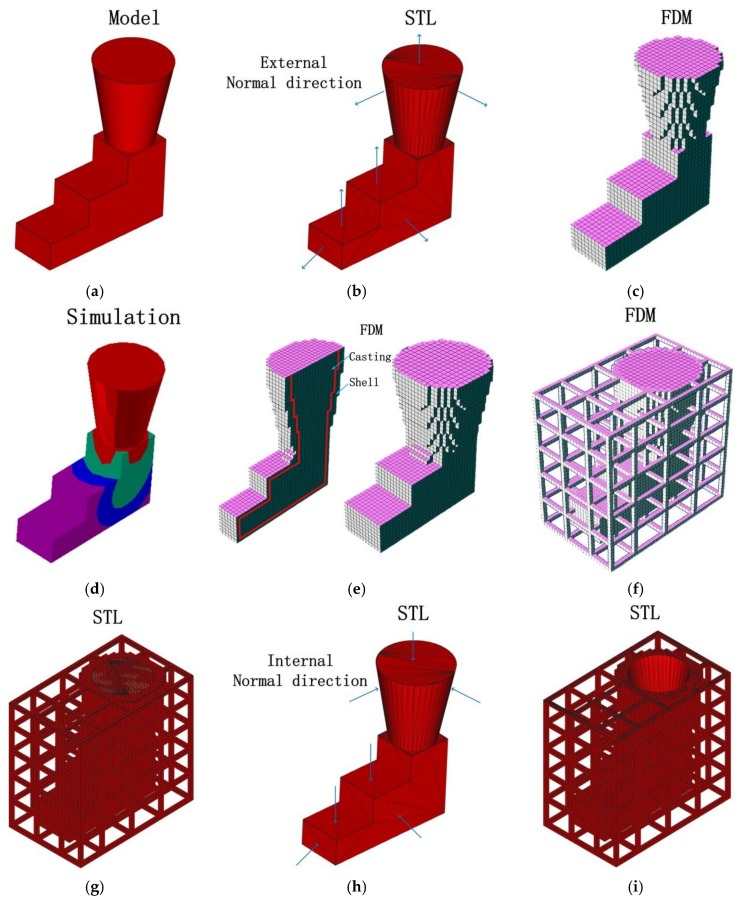
The process for generating the model based on the step-shape specimen: (**a**) 3D solid geometry; (**b**) surface discretization (STL format); (**c**) finite difference meshes; (**d**) virtual heat transfer; (**e**) shell forming with varying thickness; (**f**) lattice forming; (**g**) external surface discretization; (**h**) internal surface forming by reversing the normal of the casting surface in STL format and (**i**) lattice-reinforced thickness-varying shell mold.

In this paper, a generation method for the 3D-printed lattice-reinforced thickness-varying shell mold has been proposed, with its flowchart shown in Figure 1. The generation procedures for a mold for a step-shape specimen are shown in Figure 2. First, the STL file of a casting is exported after the construction of its 3D solid model using CAD software, which is shown in Figure 2b. The STL file is meshed into regular cubes in a finite difference mesh format (FDM), which recorded the cube lengths in x, y, z directions and the sequential number information of all the cubes (Figure 2c). After this, a shell is formed for the casting with varying thickness, which is roughly proportional to the surface temperature distribution of the casting acquired by virtually cooling it in the environment, which is shown in Figure 2d,e. Following this, the lattice structure was generated on the shell, which is shown in Figure 2f. The external surface of the finite difference meshes of the casting, shell, and lattice were transformed to an STL format file with each exposed rectangular surface split into two triangles along a diagonal, which is shown in Figure 2g. Finally, the normal directions of all of the triangles in the STL format file of the casting were reversed to form the internal surface of the new mold, which is shown in Figure 2h. The STL format file for the lattice-reinforced thickness-varying shell mold, which is shown in Figure 2i, was obtained by combining the internal surface with the external surface in STL format. The external surface of the new shell mold does not affect the shape accuracy of the casting, and thus, the size of the cubic meshes of the casting used for the shell and lattice formation is not so strictly required. Its determination should consider the calculation volume of the virtual heat transfer and the STL file size. The advantage of using the finite difference meshes of a casting is the easy proliferation of cubes in x, y, z directions and the easy discretization of the exposed surfaces of each mesh. The program was developed by C++ language to realize the automatic generation of this type of new mold for castings.

### 2.1. Thickness-Varying Shell Generation

**Figure 3:**
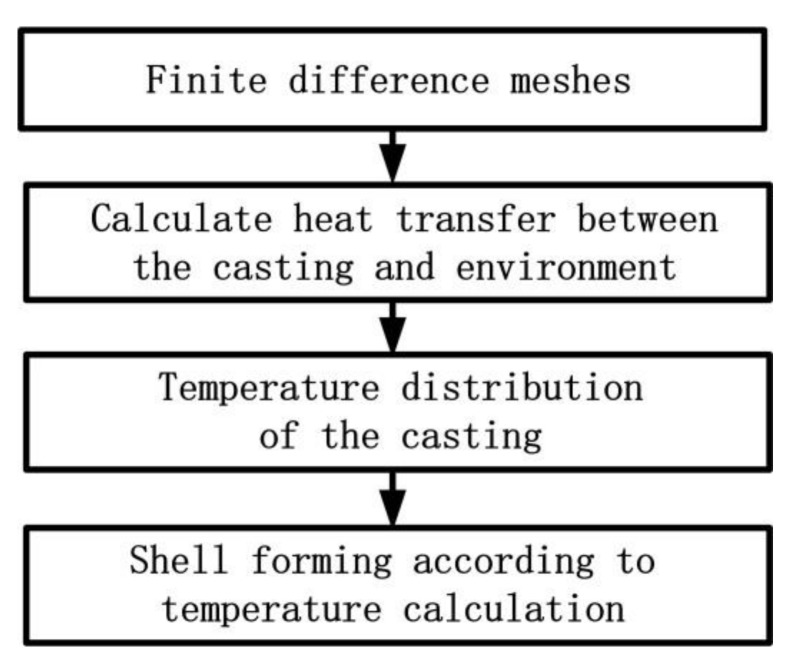
The process for generating the thickness-varying shell.

Due to the different strength requirements of thick and thin areas of a casting, the shell thickness varies along its surface. Thus, the virtual cooling of the casting in the environment can be calculated. The surface temperature distribution of the casting can reflect the thickness information. After this, the thickness of the mold shell at different locations can be generated according to the corresponding surface temperature of the casting. The process for generating the thickness-varying shell is shown in Figure 3.

The three-dimensional heat transfers satisfied the Fourier heat transfer equation as follows [20]:

(1) $${\rho c}_{p}\frac{\partial T}{\partial t}=\;\frac{\partial }{\partial x}\left(k\;\frac{\partial T}{\partial x}\right)+\;\frac{\partial }{\partial y}\left(k\;\frac{\partial T}{\partial y}\right)+\;\frac{\partial }{\partial z}\left(k\;\frac{\partial T}{\partial z}\right)$$

where ρ is the density of the casting ($\mathrm{kg}/m^{3}$); $C_{p}$ is the specific heat of the casting (J/(kg·℃)); T is the temperature of the casting (℃); t is the time of the heat transfer (s); and k is the thermal conductivity coefficient (W/(m·℃)).

The finite difference form of Equation (1) is:

(2) $$T_{i,j,k}^{\tau +1}=\frac{k}{{\rho c}_{p}}\Delta \tau \left(\frac{T_{i+1,j,k}^{\tau }-2T_{i,j,k}^{\tau }+T_{i-1,j,k}^{\tau }}{\Delta i^{2}}+\frac{T_{i,j+1,k}^{\tau }-2T_{i,j,k}^{\tau }+T_{i,j-1,k}^{\tau }}{\Delta j^{2}}\;+\frac{T_{i,j,k+1}^{\tau }-2T_{i,j,k}^{\tau }+T_{i,j,k-1}^{\tau }}{\Delta k^{2}}\right)+T_{i,j,k}^{\tau }$$

where Δτ is the time step and Δi,Δj,Δk are the step sizes for three directions.

The thickness of the shell at a certain location δ corresponds to the relevant temperature of the casting by Equation (3):

(3) $$\delta =\frac{\delta_{max}-\delta_{min}}{T_{max}-T_{min}}\left(T-T_{min}\right)+\delta_{min}$$

where $T_{max}$ is the maximum temperature (℃); $T_{min}$ is the minimum temperature (℃); $\delta_{max}$ is the maximum shell thickness (mm); and $\delta_{min}$ is the minimum shell thickness (mm). Due to the shell being composed of cubes, the shell thickness δ has to be converted into the number of layers of cubes.

### 2.2. Fillet of Rough External Surface

A sharp transition between the shell surface and the lattice structure, in addition to the bars in the lattice structure, would lead to a concentration of stress or even cracks during the casting process, so it was necessary to round up the sharp corners.

**Figure 4:**
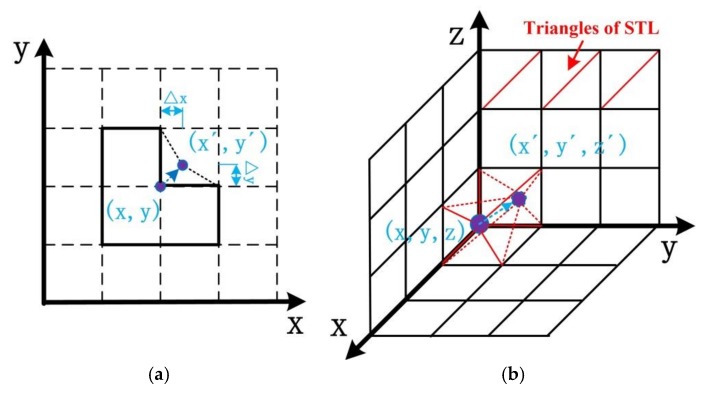
The schematic diagram of the fillet rough external surface: (**a**) groove shape; and (**b**) corner shape.

First, we found the section variation areas and corners in the shell–lattice surface according to the relationship of a cube with its adjacent neighbor cubes, based on the finite difference mesh format. There were two typical sharp transitions: groove shape and corner shape. For the groove shape, the coordinates of the nodes used in the triangles of the STL format were adjusted outward according to Equation (4), which is shown in Figure 4a.

(4) $$\{\begin{array}{c} x^{,}=x+\Delta x\\ y^{,}=y+\Delta y \end{array}$$

For the corner shape, the coordinates of the nodes used in the triangles of the STL format were adjusted by Equation (5), which is shown in Figure 4b.

(5) $$\{\begin{array}{c} x^{,}=x+\Delta x\\ y^{,}=y+\Delta y\\ z^{,}=z+\Delta z \end{array}$$

where $x^{,},y^{,},z^{,}$ are the adjusted coordinates; x,y,z are the original coordinates; and Δx,Δy,Δz are the offset values, which are less than the cube size.

### 2.3. Lattice-Structure Generation

In order to support the shell, the lattice structure should be generated around the thickness-varying shell. The lattice is in an orthogonal shape of rectangular bars composed of the basic cubes from the finite difference meshes. The section size of bars and their intervals are determined by the hydrostatic pressure of the melt.

**Figure 5:**
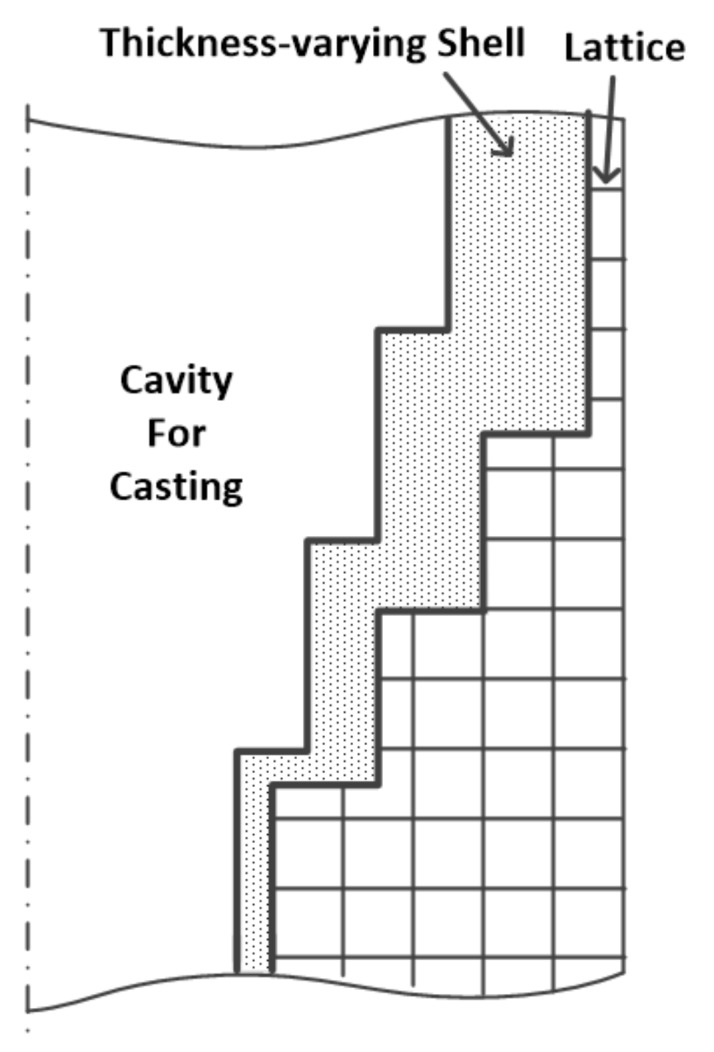
The typical lattice structure.

The typical lattice structure is shown in Figure 5.

### 2.4. Examples

**Figure 6:**
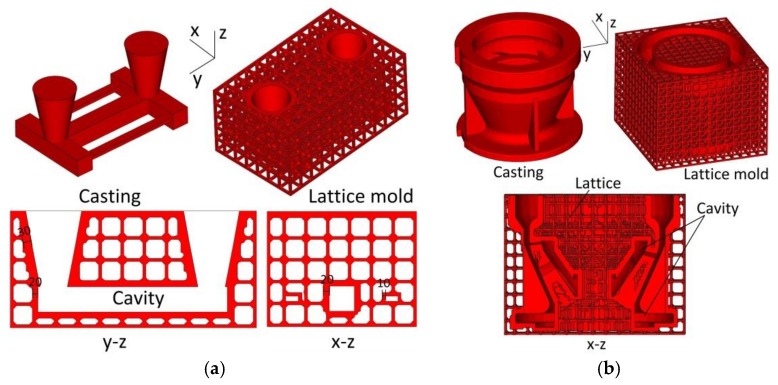
Examples of: (**a**) Stress frame casting; and (**b**) Pump bowl casting.

Two examples of the application of this generation method are shown in Figure 6: stress frame casting and pump bowl casting. Figure 6 shows that the generation method can satisfy castings of multiple risers and complex structure. The central y–z and x–z slices of the stress frame casting are shown in Figure 6a, with the thickness of the shell in the range of 10–30 mm. The central x–z section of the pump bowl casting is shown in Figure 6b.

## 3. Wheel Hub Casting Experiment Results and Discussion

**Figure 7:**
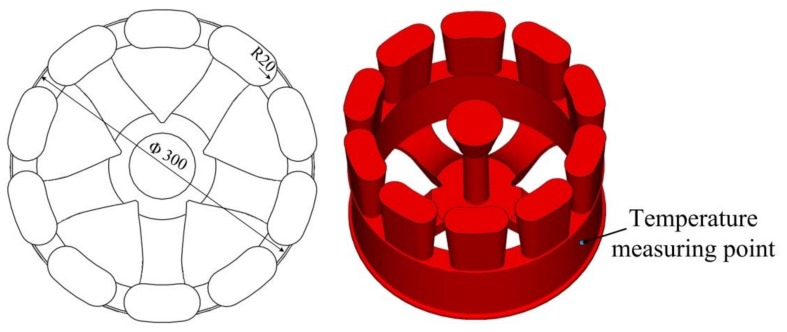
The wheel hub specimen geometry.

**Figure 8:**
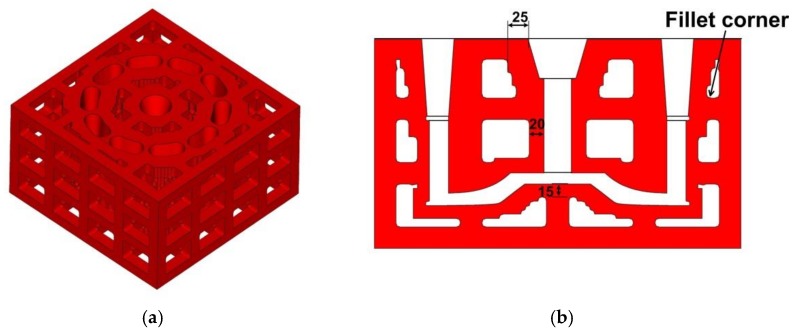
The generated lattice shell mold: (**a**) Lattice shell mold; and (**b**) Cross-section slice (Unit: mm).

The proposed lattice-reinforced thickness-varying shell structure was applied to an aluminum wheel hub casting with a diameter of 300 mm, which is shown in Figure 7. The designed mold is shown in Figure 8, which is composed of a wheel hub thickness-varying shell and an enforcing lattice.

The lattice-reinforced shell mold was printed using the ExOne-Smax 3D printing machine (ExOne, Augsburg, Germany). In 3D printing, 1.6–1.8% furan resin was used and 0.2% curing agent was added. This mold had no parting line and was composed of unit pieces. The shell was based on the thickness of casting. The range was from 15 to 25 mm.

**Figure 9:**
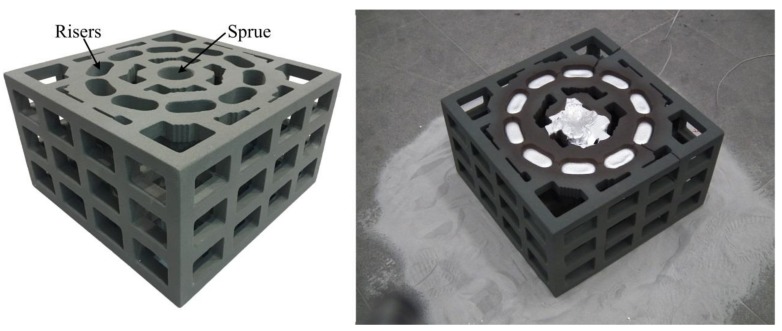
The 3D-printed lattice shell sand mold and onsite pouring experiment.

The lattices were 25 mm high and 25 mm thick with an interval of 50 mm. They are shown in Figure 9. For the convenience of sand cleaning in the mold cavity, cleaning holes were designed at positions that were otherwise difficult for sand cleaning. Furthermore, the narrow channels and cavities of the sand mold were examined with an endoscope to check the sand cleaning condition. This lattice-enforcing thickness-varying shell mold saved over 50% mold sand compared to that of the traditional dense mold.

The molten A356 aluminum alloy (composition is shown in Table 1) was poured into the mold from the sprue in the sand mold. Its liquid and solid temperature points are 616 ℃ and 556 ℃, respectively. The onsite pouring experiment is shown in Figure 9. The pouring temperature was 700 ℃ and the shakeout temperature was 200 ℃. The temperature of the wheel rim (measuring point as shown in Figure 7) was measured with the K-type sheathed thermocouple. In the casting process, the lattice enforcing the shell sand mold was cooled under natural conditions.

**Figure 10:**
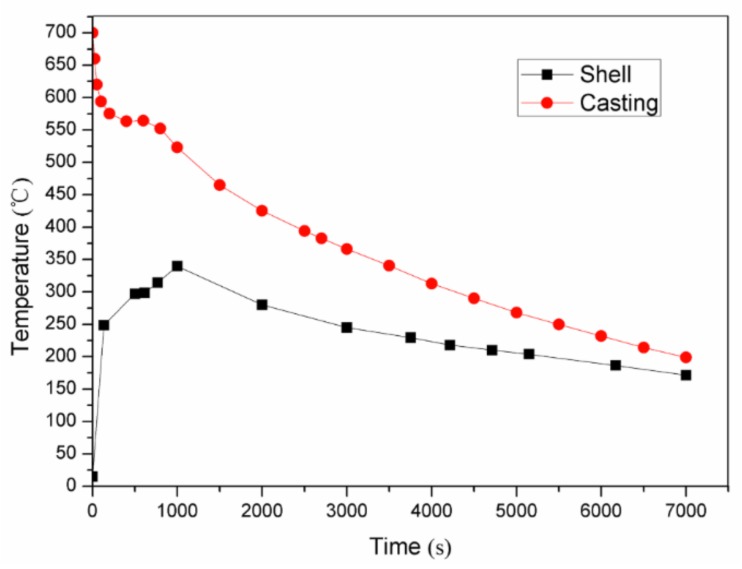
Cooling curves of the lattice-reinforced shell sand mold casting under natural conditions.

The cooling curve of the casting is shown in Figure 10. Figure 10 shows that 7000 s were needed for the cooling from the pouring temperature of 700 ℃ to the shakeout temperature of 200 ℃. The cooling efficiency of the lattice-reinforced shell sand mold was much higher than that of the traditional dense sand mold. This could increase the cooling efficiency by at least 30% for the lattice-reinforced shell sand mold casting compared to the traditional dense sand mold casting [16]. This was attributed to the fact that the lattice-reinforced shell sand mold had thin walls and a high surface temperature, resulting in high heat transfer efficiency with the surrounding air. Thus, the cooling efficiency of the lattice-reinforced shell sand mold casting was much higher.

**Figure 11:**
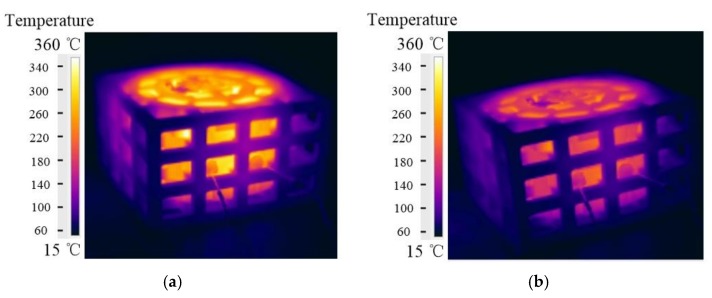
Infrared images of the lattice sand mold during the casting process: at (**a**) 1000 s; and (**b**) 5000 s.

The temperature distribution of the whole sand mold was measured using an infrared camera Flir T250 (FLIR, Wilsonville, OA, USA). Some infrared images of the lattice-reinforced shell sand mold are shown in Figure 11. After 1000 s of the pouring, the lattice-reinforced shell sand mold reached the highest temperature, which is shown in Figure 10. The highest temperature was at the shell adjacent to the riser, while the lowest temperature was observed on the lattice far away from the shell. The temperature range of lattice-reinforced shell sand mold was from 15 to 360 °C.

Based on a series of infrared images, the change in temperature variation according to time of the sand mold shell surface is plotted in Figure 10. It can be also seen that after 1000 s of pouring, the lattice shell sand mold reached the highest temperature of 340 ℃. The shell reached 300 ℃ when the casting was undergoing a solidification process. During the whole cooling process, the sand shell temperature was in the range of 170 ℃ to 340 ℃. From the cooling curve of the casting, it can be seen that the temperature variation of mold shell was increased at the beginning, before it decreased until the shakeout temperature of casting. The temperature of shell increased until the crystallization latent heat of the casting was released. When it came to the shakeout (7000 s), it was almost at 170 ℃.

**Figure 12:**
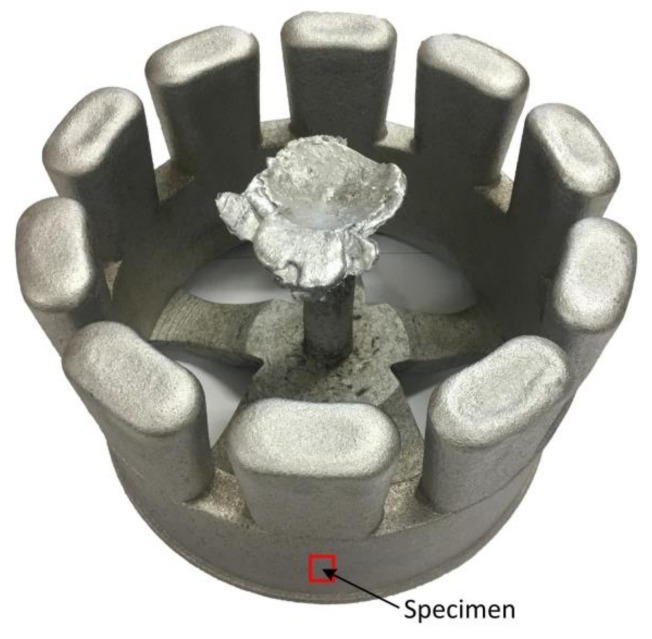
The casting obtained from the 3D-printed sand mold.

The casting obtained from the 3D-printed lattice-reinforced shell sand mold after the shakeout is shown in Figure 12. Good mold filling was observed for the casting without burr and flashing. The surface roughness of the casting was measured using the FORM TALYSURF PGI800 (Taylor Hobson, Leicseter, UK) contour graph device. It reached level four, which is better than that obtained after pouring in the traditional sand mold.

**Figure 13:**
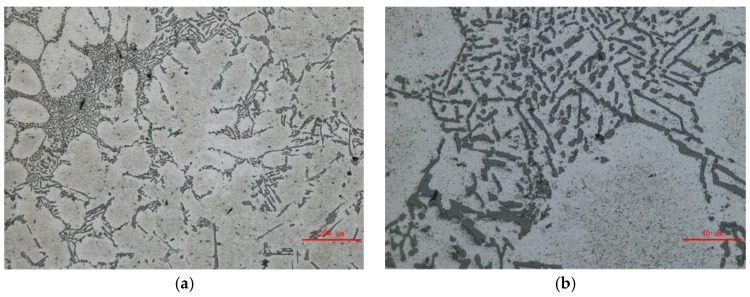
Microstructure of the casting by the lattice sand mold: (**a**) 100×; and (**b**) 500×.

The microstructure of the wheel hub casting by the lattice sand mold is shown in Figure 13. The eutectic structure was between the α-Al dendrites, with the eutectic Si being mainly globular and claviform. There was no obvious shrinkage in the casting. A specimen was cut from the casting for a hardness test, as shown in Figure 12. Its hardness was 51.3 HB.

## 4. Conclusions

(1) A generation method for a lattice-reinforced thickness-varying shell mold for 3D printing was proposed. This mold can increase the cooling efficiency of castings, realize early shakeout, and reduce sand use. It is helpful for the achievement of intelligent casting and green casting.
(2) The lattice-reinforced thickness-varying shell sand mold was successfully applied to an aluminum wheel hub casting using the proposed generation method. Good mold filling was observed. The casting occurred without burr and flashing, while its surface smoothness reached level four.

## Figures and Tables

**Table 1:** Chemical composition of A356 Al alloy (wt %).

Si	Mg	Fe	Cu	Zn	Mn	Ti	Al
6.5–7.5	0.25–0.45	0.12	0.05	0.05	0.05	0.08–0.20	Balance

## References

[1] Mami F., Rveret J.P., Fallaha S., Margni M. (2017). Evaluating Eco-Efficiency of 3D Printing in the Aeronautic Industry. J. Ind. Ecol..

[2] Levy G.N., Schindel R., Kruth J.P. (2003). Rapid manufacturing and rapid tooling with layer manufacturing technologies: State of the art and future perspectives. CIRP Ann..

[3] Miller B.W., Moore J.W., Barrett H.H. (2011). 3D printing in X-ray and gamma-ray imaging: A novel method for fabricating high-density imaging apertures. Nucl. Instr. Meth. Phys. Res. A.

[4] Liu J. (2011). The shell molding process study for mass production of single-cylinder diesel engine crankshaft. Appl. Mech. Mater..

[5] Sachs E. (1990). Three-Dimensional Printing: Rapid Tooling and Prototypes Directly from a CAD Model. ClRP Ann..

[6] Michael F., Ciaran M., Jonathan B., Erik H., James M., Michael O. (2014). Automatic innovative truss design using grammatical evolution. Autom. Constr..

[7] Dutta B., Froes F.H., Qian M., Froes F.H. (2015). Titanium Powder Metallurgy: Science Technology and Applications.

[8] Wood K., Ravi S. Design considerations for three dimensional printed cores and molds. Proceedings of the 119th Metal Casting Congress.

[9] Meet U., Tharmalingam S., Mohamed E.M. (2017). 3D printing for rapid sand casting—A review. J. Manuf. Process..

[10] Thomas B., John U. (2013). 3D, SF and the future. Futures.

[11] Kang J.W., Qiangxian Ma Q.X. (2017). The role and impact of 3D printing technologies in casting. China Foundry.

[12] Deng C.Y., Kang J.W., Shangguan H.L., Hu Y.Y., Huang T. (2018). Effects of hollow structures in sand mold manufactured using 3D printing technology. J. Mater. Process. Technol..

[13] Kirleis M.A., Simonson D., Charipar N.A. (2014). Laser embedding electronics on 3D printed objects. Proc. SPIE.

[14] Kang J.W., Shangguan H.L., Deng C.Y. (2017). New Mold of Non-Dense Structure. CHN Patent.

[15] Kang J.W., Shangguan H.L., Hu Y.Y. (2018). New Mold of Double Shell Structure. CHN Patent.

[16] Shangguan H.L., Kang J.W., Deng C.Y., Hu Y.Y., Huang T. (2017). 3D-printed shell-truss sand mold for aluminum castings. J. Mater. Process. Technol..

[17] Alok K.P., Satyandra K.G. (2004). Geometric algorithms for automated design of multi-piece permanent molds. Comput. Aided Des..

[18] Chen Y. (2007). 3D Texture Mapping: A Microstructure Design Method for Rapid Manufacturing. Comput. Aided Des..

[19] Chen Y., Wang S.L. (2008). Computer-aided Product Design with Performance-Tailored Mesostructures. Comput. Aided Des..

[20] Zhang X.M., Ren Z.L., Mei F.M. (2007). Heat Transfer.

